# The liver, a functionalized vascular structure

**DOI:** 10.1038/s41598-020-73208-8

**Published:** 2020-10-01

**Authors:** Sylvie Lorente, Mathieu Hautefeuille, Aczel Sanchez-Cedillo

**Affiliations:** 1 Department of Mechanical Engineering, Villanova University, Villanova, PA 19085 USA; 2 Departamento de Física, Facultad de Ciencias, Universidad Nacional Autónoma de México, Circuito Exterior S/N, Ciudad Universitaria, CP04510 Coyoacán, Ciudad de México, Mexico; 3 Centro Médico 20 de Noviembre, ISSSTE,, Félix Cuevas 540, Del Valle Sur, Benito Juárez, CP03100 Ciudad de México, Mexico

**Keywords:** Engineering, Liver

## Abstract

The liver is not only the largest organ in the body but also the one playing one of the most important role in the human metabolism as it is in charge of transforming toxic substances in the body. Understanding the way its blood vasculature works is key. In this work we show that the challenge of predicting the hepatic multi-scale vascular network can be met thanks to the constructal law of design evolution. The work unveils the structure of the liver blood flow architecture as a combination of superimposed tree-shaped networks and porous system. We demonstrate that the dendritic nature of the hepatic artery, portal vein and hepatic vein can be predicted, together with their geometrical features (diameter ratio, duct length ratio) as the entire blood flow architectures follow the principle of equipartition of imperfections. At the smallest scale, the shape of the liver elemental systems—the lobules—is discovered, while their permeability is also predicted. The theory is compared with good agreement to anatomical data from the literature.

## Introduction

The liver is one of the most important organ of the human body. Its role is fundamentally important, particularly in the transformation of toxic substances to elements that the body can eliminated. The liver vasculature makes its unique among the other organs as it is made of the superimposition of three main networks, two inlets and one outlet. The two inlet networks, namely the hepatic artery (HA) and the portal vein (PV), run in parallel. The hepatic artery provides oxygenated blood to the liver, while the portal vein brings deoxygenated blood; the two mix in the sinusoids. The sinusoids are uniformly distributed throughout the entire liver volume, and constitute the hepatic microcirculation. The latter is a three-dimensional network forming a lattice between rows cells, mainly hepatocytes, epithelial cells responsible for the metabolism taking place in the liver. The sinusoids together with the hepatocyte cords form the smallest units of the liver: the lobules. The lobule is considered to be the liver functional unit, the elemental system. The lobules are represented classically by prismatic volumes of hexagonal cross section with one triad made of the hepatic artery, the portal vein and the bile duct at each of the 6 hexagon corners, and a central vein along the axis of the lobule. The central veins, or hepatic veins (HV) collect the blood and lead it to the vena cava inferior.

The modeling of the hepatic circulation has been a challenge for several decades. Our approach is based on the constructal law of design^1–4^. Back in the mid twentieth century, Landes^5^ noticed in 1943 the analogy between blood flows and electrical currents transport. Models range from the entire circulatory architecture^6^ to the hepatic micro circulation^7,8^. Virtual liver networks combine biology to fluid mechanics and mass transfer as novel approaches to physiology models^9–13^. However these models, regardless their sophistication level^14,15^, are descriptive. Missing is a theoretical approach, based on first principles, that would allow to predict the flow architecture of the hepatic circulation: liver transplant, or liver resection as a treatment for liver tumors, may end up to liver failure with disastrous consequences when the change in blood pressure is not controlled^14–16^. Indeed, Fisher^17^ in 1954 showed experimentally that the volume of blood reaching the liver and somehow the "delivered pressure", have an evident impact on the regenerative stimulus of liver regeneration with 3 surgical variants of restitution of portal flow plus hepatectomy. Understanding and controlling the liver portal pressure after surgery would be of the utmost importance to guarantee correct regeneration signals and prevent cell death^18^.

As indicated by precise anatomy analysis of the liver architecture^19–22^, the hepatic artery, portal vein and hepatic vein networks are three-dimensional, highly nested, forming an extremely compact structure. Their complexity often forces to reduce the hydrodynamic studies of the liver to its morphofunctional unit, the lobule^23,24^. Considering any of the 3 networks, we note that each consists in a tree-shaped architecture delivering the blood through the connection of one inlet to an infinite number of outlets, the lobules (case of the hepatic artery and the portal vein), or from the lobules to one outlet in the case of the HV hepatic venous system. Tree-shaped architectures are configurations that correspond to a point-to-volume flow. For a long time, such structures were considered as being the result of chance, i.e. non deterministic. Yet, about 20 years ago, A. Bejan^25^ proved that dendritic configurations can be predicted, and that they exist for a very specific purpose. Wechsatol et al.^26^ showed that they happen because they are the most efficient way, i.e. with minimum work, to deliver a fixed volume of fluid from one point to an infinite number of points (a volume or a surface). Incidentally, the deterministic structure of the body fluid networks was highlighted in the early twentieth century by respectively Hess^27^ and Murray^28^ who came to the conclusion that the diameter ratio between mother and daughter branches has a unique value.

The objective of this work is to lay the theoretical foundations of the deterministic behavior of the human liver vasculature, as a hierarchical fluid mechanics system. The work relies on the fundamentals of fluid mechanics and allows to predict how the blood transport happens through a human liver. In our approach, the liver becomes a multi-scale dendritic fluid network constituted of superimposed tree-shaped architectures that provides and drains the blood flow.

## The dendritic architecture of the liver blood network

Detailed measurements of the geometrical features of the human liver blood network are scarce. To confront the theoretical approach developed in this paper to experimental data, we rely on anatomic results provided by two different groups^19,22,29^. The authors published the radii, lengths and number of branches of the hepatic artery, the portal vein and the hepatic vein. The three vascular architectures constitute the macro and meso circulation of the human liver. Debbaut et al.^19,29^ explained that they could not measure the total number of branches when the splitting level increases. Therefore, from the measurements of 4 representative portions of the liver, they considered that the total number of branches could be estimated in proportion of the total liver volume. Ma’s group^22^ managed to measure up to the 20^th^ generation for the 3 different vascular networks, and reported the channels diameters. Table 1 provides the diameter ratio, the channel length ratio and the splitting number at each generation number. The splitting number is calculated from the ratio of the number of daughter branches and mother branches. The average values together with their standard deviation are also provided. On an average, the measured splitting number is 2.76 for the hepatic artery, 2.80 for the portal vein, and 3.22 for the hepatic vein, which translated into the integer $n$ = 3.

**Table 1:** Calculations of the diameter ratio, length ratio and splitting number of branches, data from Debbaut et al.^19,29^ and Ma et al.^22^.

Generation number	Debbaut et al.^19,29^									Ma et al.^22^		
	Hepatic Artery, HA			Portal Vein, PV			Hepatic Vein, HV			HA	PV	HV
	$\frac{{d}_{i+1}}{{d}_{i}}$	$\frac{{L}_{i+1}}{{L}_{i}}$	Splitting number	$\frac{{d}_{i+1}}{{d}_{i}}$	$\frac{{L}_{i+1}}{{L}_{i}}$	Splitting number	$\frac{{d}_{i+1}}{{d}_{i}}$	$\frac{{L}_{i+1}}{{L}_{i}}$	Splitting number	$\frac{{d}_{i+1}}{{d}_{i}}$		
1	0.83		2.0	0.86	1.00	2.0	0.38	1.09	3.0	0.95	0.86	0.57
2	0.62	0.54	3.5	0.63	0.94	3.5	0.48	0.45	6.0	0.92	0.98	1.00
3	0.61	0.78	5.1	0.53	0.97	3.9	0.71	0.85	1.8	0.96	0.82	1.00
4	0.75	0.81	3.3	0.71	0.84	3.0	0.65	0.68	2.3	1.00	0.99	0.92
5	0.71	0.64	2.3	0.59	0.55	2.8	0.67	0.45	2.3	0.72	0.80	0.88
6	0.58	0.45	2.0	0.81	0.55	2.0	0.62	0.77	2.9	0.50	0.99	0.61
7	0.80	0.49	2.8	0.67	0.64	3.1	0.54	0.62	4.3	0.75	0.43	0.91
8	0.87	0.88	2.2	0.64	0.60	2.8	0.70	0.64		0.88	0.74	0.30
9	0.86	0.76	2.6	0.75	0.85	2.8				0.58	0.90	0.93
10	0.77	0.63	1.7	0.76	0.55	2.3				0.93	0.74	0.79
11	0.74	0.68		0.79	0.70					0.63	0.66	0.85
12										0.81	0.59	0.79
13										0.61	0.67	
14										0.65	0.89	
15										0.96		
16										0.87		
17										0.83		
18										0.63		
19										0.90		
20										0.67		
Average	0.74	0.66	2.76	0.70	0.72	2.80	0.59	0.64	3.22	0.79	0.79	0.80
σ*	0.10	0.14	1.01	0.10	0.18	0.61	0.12	0.21	1.46	0.15	0.16	0.21

*σ is the standard deviation.

### Diameter ratio

Consider the laminar steady flow of a fluid through a dendritic network. In the simplest case we assume that the ducts have a round cross section of diameter *d* and length *L*. The pulsatile nature of blood flow in the hepatic artery network is classically expressed through an electrical impedance analogy. This does not have impact in the theoretical approach presented here because the frequency domains relevant to biological flows, as in the case of the liver, correspond to a negligible imaginary contribution in the impedance expression of the fluid flow, leaving a direct proportionality between pressure difference and mass flow rates as provided by the real part of the impedance^30^. Blood is a non-Newtonian fluid. From a rheological viewpoint, blood belongs to the class of shear shinning fluids, also called pseudo-plastic, as its dynamic viscosity decreases when the shear strain increases. As such, it is often described by a power-law model. Nevertheless, and following the work of Revellin et al.^31^, Hess-Murray’s law remains valid even with a power-law model.

In accord with the constructal law of design^32^, fluid architectures evolve in time to facilitate access to the currents flowing through them, for minimum pumping power. We are interested in predicting the geometrical features leading to the smallest overall pressure difference possible. The fluid enters a mother tube (diameter ${d}_{i}$ and length ${L}_{i}$) and splits through *n* daughter tubes of constant geometrical features (diameter ${d}_{i+1}$ and length ${L}_{i+1}$). The total blood volume *V* is fixed, so is the mass flow rate of blood. For the blood to flow through the entire body, a pump is needed. The necessary pumping power is provided by the heart, and is proportional to $\dot{m}\times \Delta p$. Based on the previous assumptions, the friction losses and total volume vary as

1 $$\Delta p \sim \dot{m}\frac{{L}_{i}}{{d}_{i}^{4}}+\frac{\dot{m}}{n}\frac{{L}_{i+1}}{{d}_{i+1}^{4}}$$

2 $$V \sim {d}_{i}^{2}{L}_{i}+n{d}_{i+1}^{2}{L}_{i+1}$$

The minimum pressure difference is obtained by minimizing $\Delta p$ for the fixed volume $V$. The corresponding diameter ratio is given by^31^:

3 $$\frac{{d}_{i+1}}{{d}_{i}}={n}^{-1/3}$$

As the splitting number is $n$ = 3 on an average, Eq. 3 predicts that the corresponding diameter ratio should be 3^–1/3^ ≅ 0.69 in order to minimize the pumping power needed to push the blood in and out the liver.

This result is in good agreement with the anatomical data. We wrote in Table 1 the diameter ratios at each generation level, their average value and standard deviation σ for the 3 flow configurations, calculated from the data provided in Refs. 16, 21 and 28. The results are ${d}_{i+1}/{d}_{i}=$ 0.74, 0.70, and 0.59 for the hepatic arteria, the portal vein and the hepatic vein respectively according to Debbaut et al.^19,29^, while a ratio of 0.79 is obtained from the measurements provided by Ma et al.^22^.

### Channel length ratio

For each network to be fully determined, we also need to predict the tube lengths ratio, and prove the merit of a dendritic-based architecture as opposed to a radial fluid distribution. If $g$ is the generation number ($g$ may vary from one tree to another) than the total number of HA (or PV) outlets or HV inlets is ${n}^{g}= {3}^{g}$, with a splitting number *n* = 3.

The flow resistance created in the case of a dendritic design is given by

4 $${\left.\frac{\Delta p}{\dot{m}}\right)}_{dendritic}\sim \frac{{L}_{1}}{{d}_{1}^{4}}+\frac{1}{3}\frac{{L}_{2}}{{d}_{2}^{4}}+\frac{1}{{3}^{2}}\frac{{L}_{3}}{{d}_{3}^{4}}+\dots +\frac{1}{{3}^{g-1}}\frac{{L}_{g}}{{d}_{g}^{4}}$$

In their 2005 paper, Wechsatol et al.^33^ documented the design of laminar dendritic networks on a fixed disc-shaped area. The objective was to connect one inlet at the center of the disc to a large number of points distributed on the disk perimeter. The architecture was based on bifurcation patterns. The network was completely determined thanks to (i) the radius ratio between a daughter branch and a mother branch in accord with the Hess-Murray’s law (${d}_{i+1}/{d}_{i}={2}^{-1/3}$, where 2 was the number of daughter branches), and (ii) the connection angles between ducts at each bifurcation level. The latter led to the duct length ratio. The study indicated that the tube length ratio (daughter tube length divided by mother tube length) for minimum pumping power was a constant of about 0.50 regardless the number of tubes connected to the center of the disk. Relying on these findings, we write ${L}_{i+1}=k{L}_{i}$, where k is to be discovered. Here we do not consider the very first tube length ratio as no information is provided on the first duct length in Refs^19,29^. Combining Eq. 4 with the definition of the fluid volume ${V \sim {d}_{1}^{2}L}_{1}\sum_{i=0}^{g}{3}^{i/3}{k}^{i}$, we finally write:

5 $${\left.\frac{\Delta p}{\dot{m}}\right)}_{dendritic} \sim \frac{{L}_{1}^{3}}{{V}^{2}}{\left[\sum_{i=0}^{g}{\left({3}^{1/3}k\right)}^{i}\right]}^{3}$$

We demonstrated previously that the most efficient flow architectures are the ones featuring equipartition of thermodynamics imperfections^32^. In the case of fluid flow networks it means that the minimum pumping power is found when the pressure losses are equally distributed over the flow architecture. Detailed examples can be found in Refs^34–36^. In the paper published by Miguel^35^ in 2016, the equipartition of thermodynamics imperfections concept translates into an equipartition of flow resistances. In Eq. 5 this means that ${3}^{1/3}k$ = 1, or said in other words:

6 $$\frac{{L}_{i+1}}{{L}_{i}}={3}^{-1/3}$$

and

7 $${\left.\frac{\Delta p}{\dot{m}}\right)}_{dendritic} \sim \frac{{L}_{1}^{3}}{{V}^{2}}{\left(g+1\right)}^{3}$$

**Figure 1:**
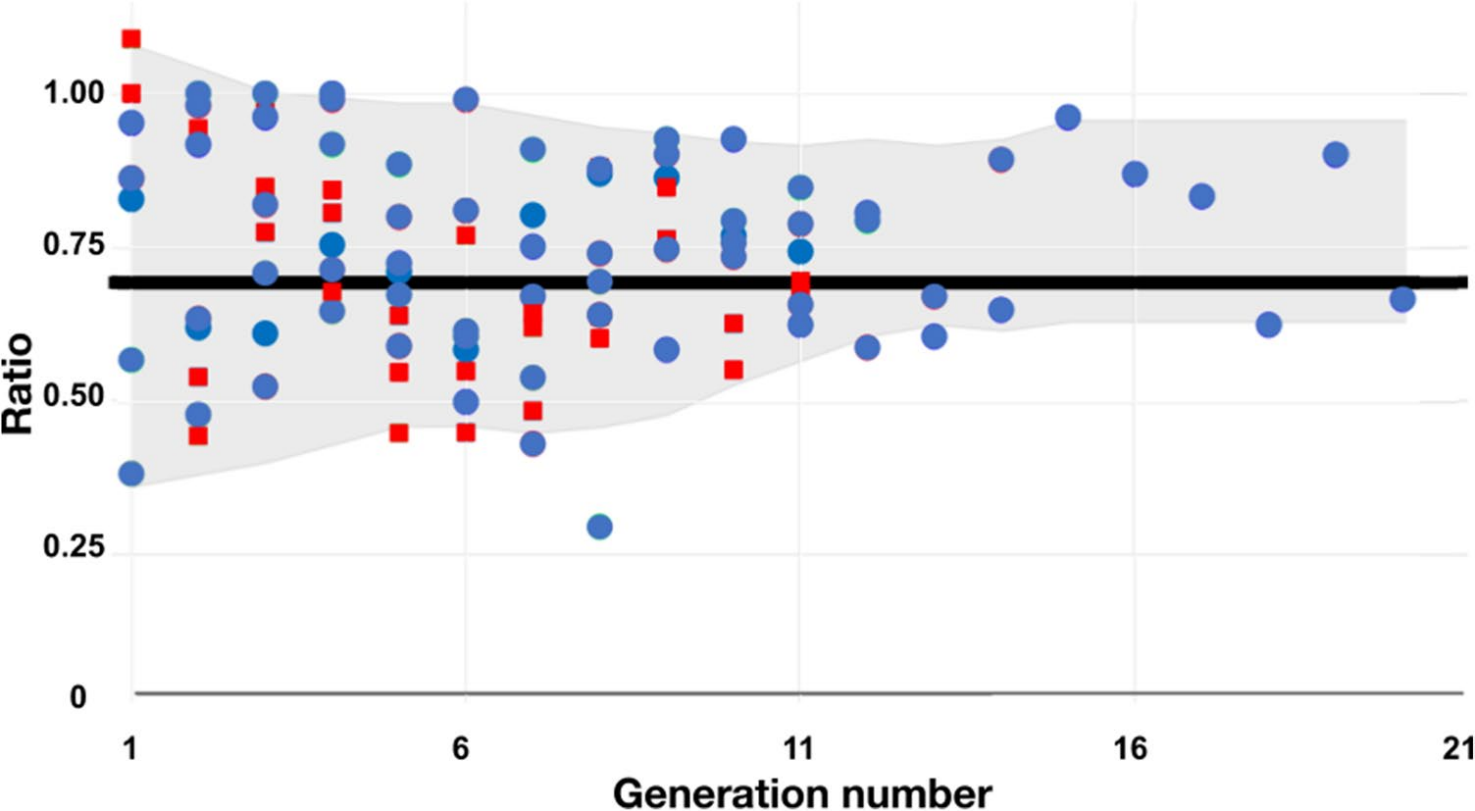
The measured diameters (*blue dots*) and lengths (*red squares*) ratios inside the liver as a function of the splitting generation number, for the hepatic artery HA, the portal vein PV and the hepatic vein HV. The data are from Refs^16,21,28^.

The averaged measured channel length ratio is 0.66, 0.72 and 0.66 for respectively HA, PV and HV. Accounting for the discrepancy of the measurements from one generation level to the other, we consider the predicted value of ${3}^{-1/3}\cong$ 0.69 as reliable. Note that the predicted tube length ratio is identical to the channel diameter ratio. The results are gathered in Fig. 1 for a bird-eye view.

### Why dendritic?

**Figure 2:**
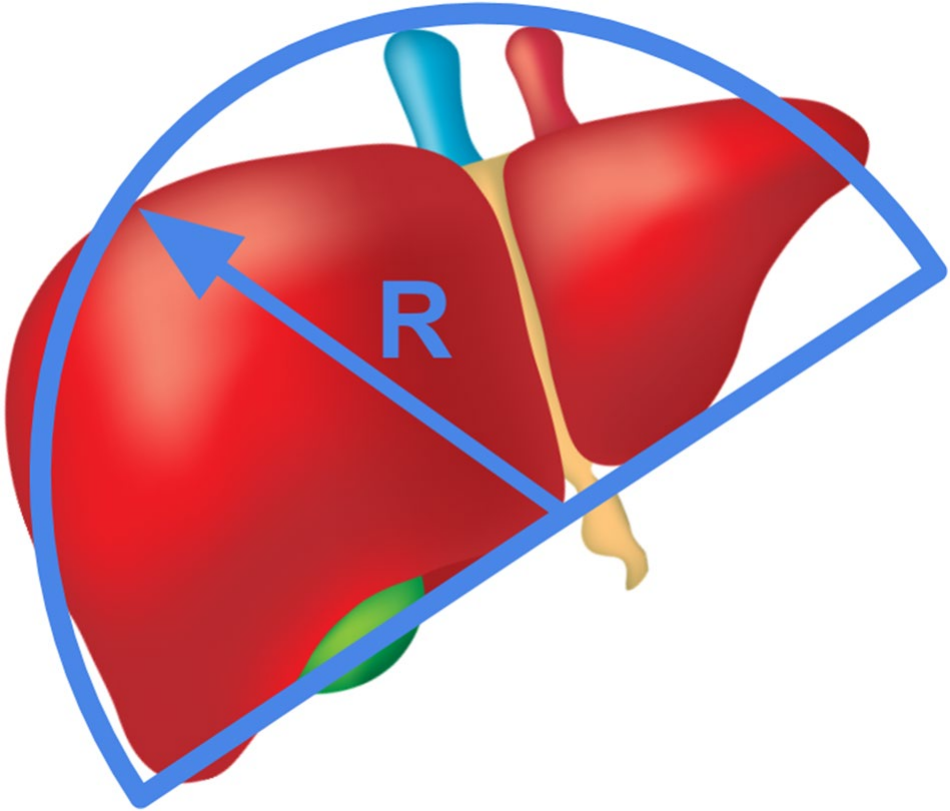
The shape of the liver as a hemisphere of radius *R.*

Once the geometrical ratios that characterize the liver vasculature are uncovered, the merit of a dendritic flow architecture over a radial one can be checked. The shape of the liver is assumed to be a hemisphere with a radius R, the center of which being located at the level of the blood inlet (Fig. 2). The radial distribution of the fluid would generate a flow resistance ${\left.\Delta P/\dot{m}\right)}_{radial}$ which order of magnitude is $R/{3}^{g}{d}_{radial}^{4}$. In the radial distribution configuration, the unique diameter of each duct is known because the total fluid volume in each tree network is fixed and is such that $V={3}^{g}\frac{\pi }{4}{d}_{radial}^{2}R$. Therefore, ${\left.\Delta P/\dot{m}\right)}_{radial}$ scales as ${3}^{g}{R}^{3}/{V}^{2}$.

By the same token, the flow resistance ratio between the dendritic and radial configurations is:

8 $${{\left. {\frac{{\Delta p}}{{\dot{m}}}} \right)_{{dendritic}} } \mathord{\left/ {\vphantom {{\left. {\frac{{\Delta p}}{{\dot{m}}}} \right)_{{dendritic}} } {\left. {\frac{{\Delta p}}{{\dot{m}}}} \right)_{{radial}} \sim\left( {\frac{{L_{1} }}{R}} \right)^{3} \frac{{(g + 1)^{3} }}{{3^{g} }}}}} \right. \kern-\nulldelimiterspace} {\left. {\frac{{\Delta p}}{{\dot{m}}}} \right)_{{radial}} \sim\left( {\frac{{L_{1} }}{R}} \right)^{3} \frac{{(g + 1)^{3} }}{{3^{g} }}}}$$

where ${L}_{1}$ is the channel length corresponding to the first generation number.

The term ${(g+1)}^{3}/{3}^{g}$ is lower than 1 as soon as the generation number reaches a value of 5, while ${L}_{1}<R$. In conclusion, ${\left.\Delta p/\dot{m}\right)}_{dendritic}\ll {\left.\Delta p/\dot{m}\right)}_{radial}$. In the case of the tree networks that compose the liver vascular system, the generation number is about 20. In a point-to-volume configuration the tree-shaped architecture exists because it is endowed with less friction losses compared to a radial flow distribution, just like in the river delta analogy.

In conclusion, each blood architecture corresponds to a system allowing a point-to-volume (or volume-to-point) fluid distribution for minimum pumping work.

## Microcirculation, the lobules

**Figure 3:**
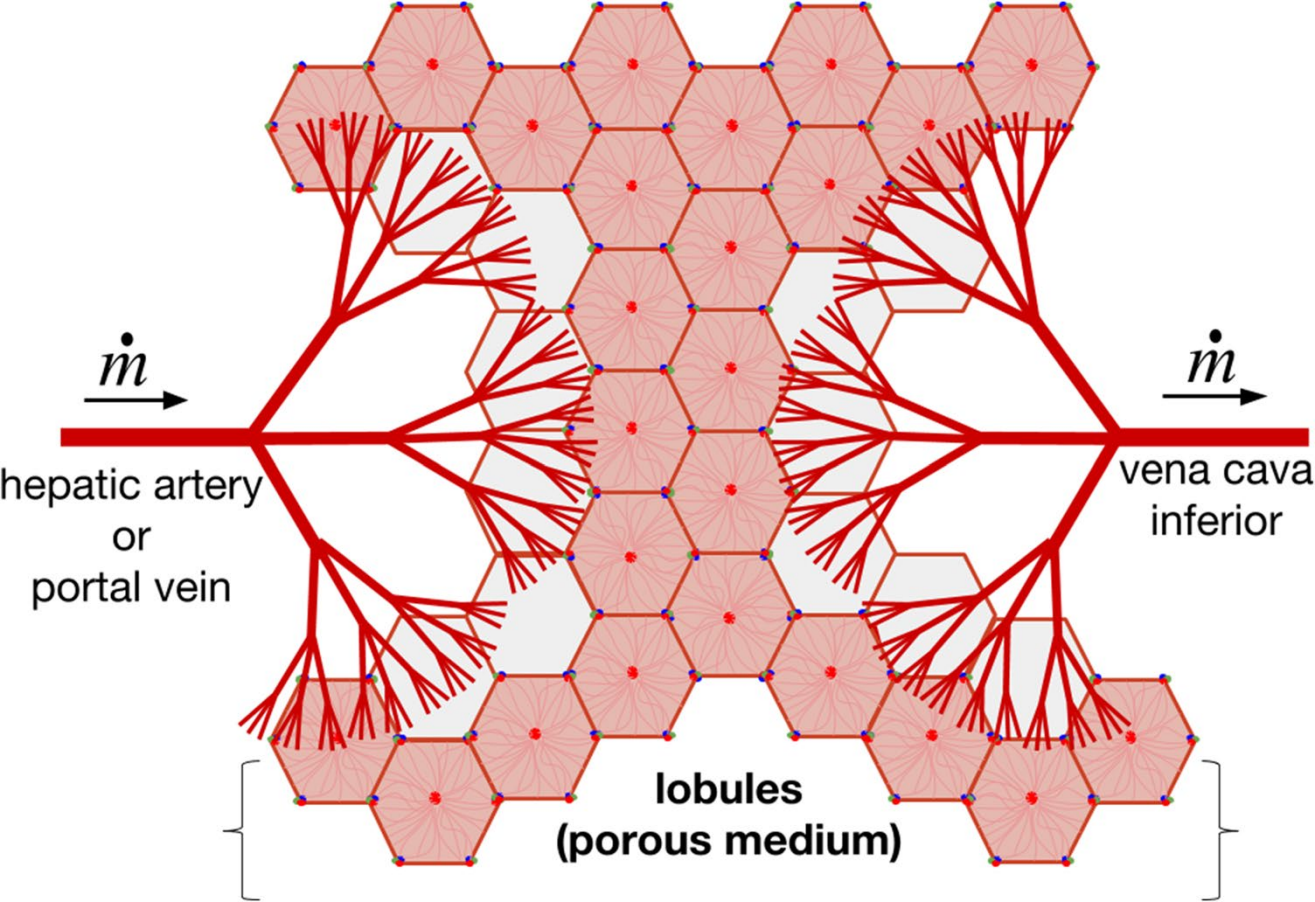
The canopy-to-canopy view of the liver vascular system. The inlet vasculature is made of the hepatic artery and the portal vein, the outlet vasculature is made of the hepatic vein. The lobules area porous medium located between the tree canopies.

The hepatic artery, portal vein and hepatic vein form a compact three-dimensional dendritic architecture within the liver. Each tree architecture is composed of a main trunk subdivided into smaller and smaller braches. The thinner channels form the canopy of the HA and PV trees and irrigate the lobules which behave like a porous system. Further downstream, the flow from the two inlet trees is reconstituted into a single stream through the outlet HV tree. Conceptually, the liver vascularization can be seen as 2 trees matching canopy-to-canopy bathing a porous architecture made of lobules, as presented in Fig. 3. Configurations of trees matching canopy-to-canopy were already presented by our group in the context of engineering applications^37–39^. We showed that the global flow resistance decreases as the number of bathed elements connected to the trees increases.

Should the objective be to flow from the gastrointestinal tract to the inferior vena cava and the heart, then the straight channel would be the configuration that we should see. On the contrary, the blood flowing in reaches first the smallest liver units, the lobules, before flowing out through the hepatic veins and then the vena cava inferior. The assembly of lobules constitutes a functionalized porous medium which must allow the mixing of the oxygenated blood from the hepatic artery and the deoxygenated blood from the portal vein*.* The hepatic artery brings oxygenated blood. The portal vein brings blood rich in nutrients and antigens from the gastrointestinal system. Both sources of blood mix in the sinusoids. All the cells of the porous lobule-system fulfill the metabolic and filtering functions. Once mixed the blood is pushed into the hepatic vein.

### The shape of the elemental system

**Figure 4:**
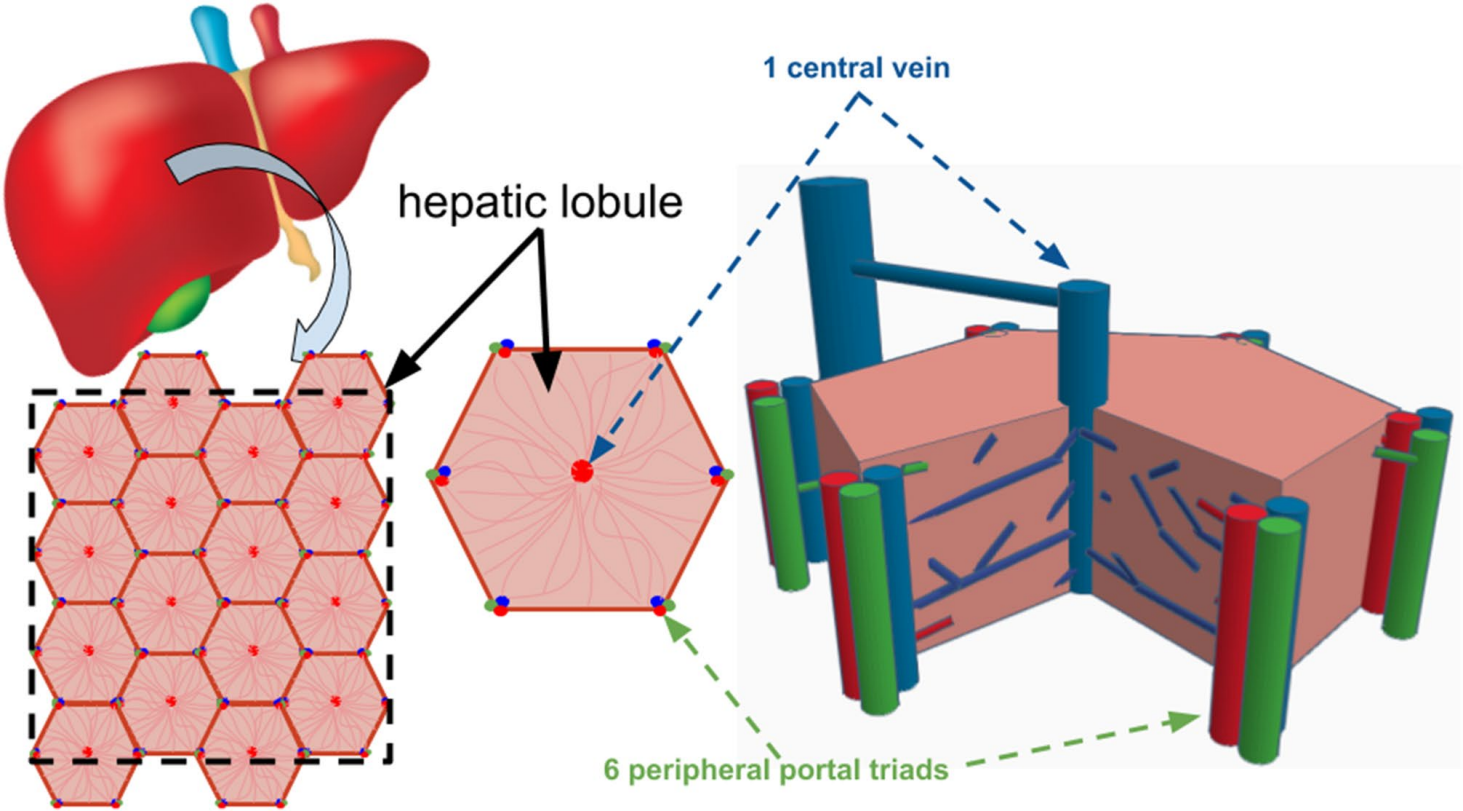
Lobules of the liver paving the entire available domain. The inlet flow comes from the hepatic artery and the portal vein constituting the triad (the third element being the bile canal). The outlet flow (hepatic vein) is located at the center of each lobule.

The lobules which constitute the designed porous medium have a highly regular design. Figure 4 depicts cross sections of them, and gives an overview of one single lobule, the elemental system. The hexagonal cross section shows 6 portal triads made of the bile duct, the portal vein and the hepatic artery. Here we do not consider the bile canal. The blood mixes along each of the sinusoids, which are mainly perpendicular to the portal triad. Blood is then pushed out when reaching the center of the lobule through the central vein parallel to the portal triad. Describing the lobules under the assumption of slices of highly vascularized hexagons represents a commonly admitted hypothesis.

**Figure 5:**
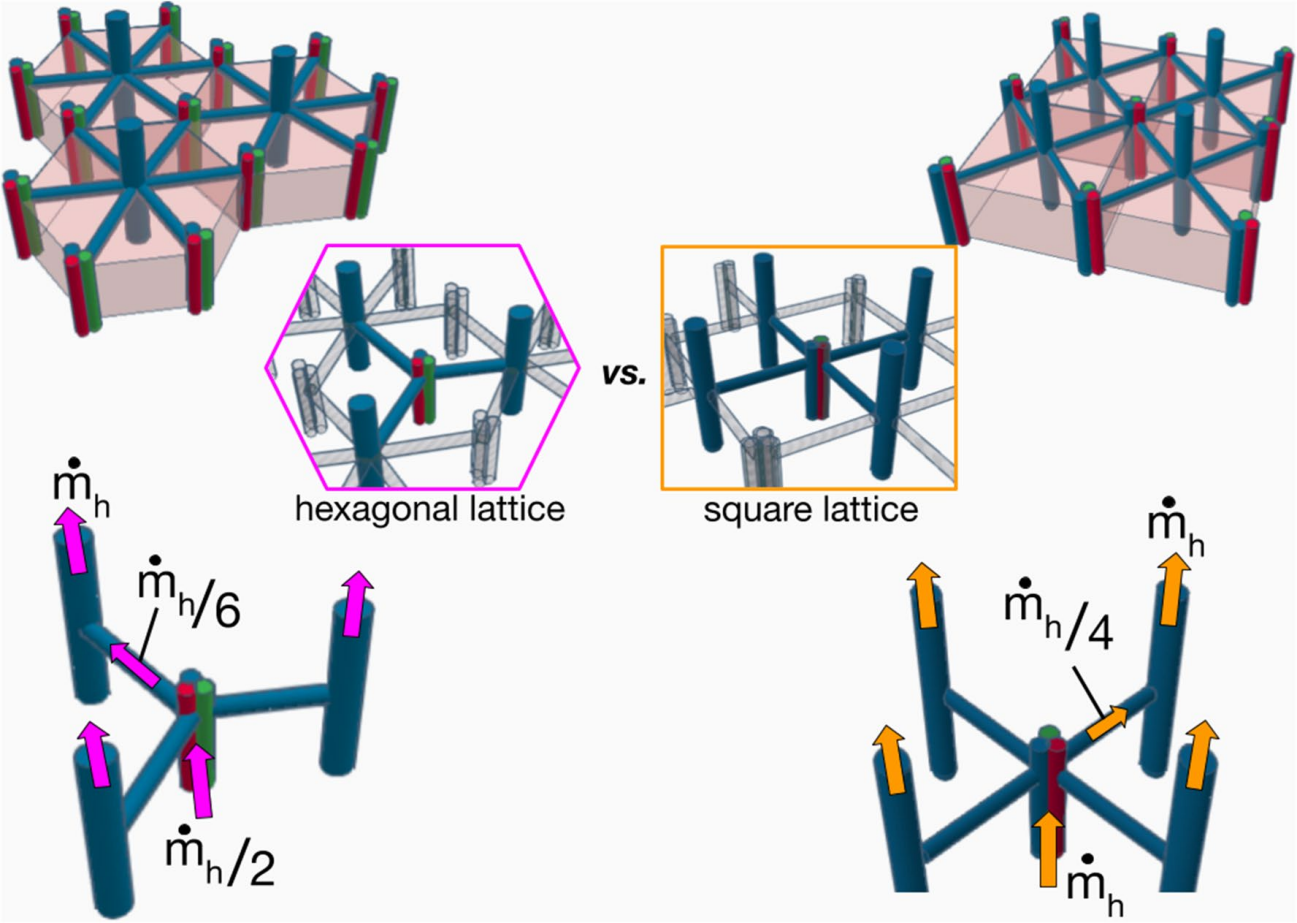
Sketch of hexagonal (left side) and square configurations (right side). The blood flow path is represented by the arrows.

Why a hexagon? If we look into Fig. 4, we see that the square image is made of about 16 hexagonal shapes of side ${L}_{h}$. Calling A_h_ the area of the hexagon, we have ${A}_{h}=\frac{3\sqrt{3}}{2}{L}_{h}^{2}$. The mass flow rate that exits one hexagon is ${\dot{m}}_{h}$, and therefore the total mass flow rate through the square delimited by the dashed lines would be ${16 \dot{m}}_{h}$. Each central vein of a hexagon receives ${\dot{m}}_{h}/$6 from the 6 triads distant of ${L}_{h}$ from the central vein (the hexagon is made of 6 equilateral triangles of side ${L}_{h}$). Assume one main sinusoid of diameter ${d}_{h}$ connects a triad to the central vein. The assembly of hexagonal shapes makes each hepatic artery and portal vein in contact with 3 lobules. For the sake of simplicity, assume that the hepatic artery and the portal vein are one single conduct of diameter $d$ and length ${L}_{d}$, through which the mass flow rate is hence $3{\dot{m}}_{h}/6$. The flow path is represented on the left hand side of Fig. 5.

The total pressure difference between the inlet and the outlet of a lobule is given by

9 $${\Delta p}_{t,h} \sim \frac{{\dot{m}}_{h}}{2}\frac{{L}_{d}}{{d}^{4}}+\frac{{\dot{m}}_{h}}{6}\frac{{L}_{h}}{{d}_{h}^{4}}$$

Another way to pave entirely the square domain represented in Fig. 4 would be to use squared shaped lobules of side ${L}_{S}$ with ${A}_{S}={L}_{S}^{2}$. We consider that ${A}_{h}={A}_{S}$, which means that the hexagon is reshaped to become a square, and ${L}_{S}=\frac{{3}^{3/4}}{\sqrt{2}}{L}_{h}\cong 1.6{L}_{h}$. The total number of squares is the same as the number of hexagons, namely 16. Therefore, because the total mass flow rate is a constant, the mass flow rate per element must continue to be ${\dot{m}}_{h}$. Each square element receives the blood from each of its 4 corners. This means that in this configuration, the central vein would be connected to 4 radial branches of diameter ${d}_{c}$ and length ${L}_{c}=\sqrt{2}{L}_{S}$ through which the mass flow rate is ${\dot{m}}_{h}/4$. As each square element is in contact with 3 other ones, the mass flow rate through the duct of diameter $d$ and length ${L}_{d}$ must be ${\dot{m}}_{h}$. Finally the overall pressure loss is

10 $${\Delta p}_{t,c} \sim {\dot{m}}_{h}\frac{{L}_{d}}{{d}^{4}}+\frac{{\dot{m}}_{h}}{4}\frac{{L}_{c}}{{d}_{c}^{4}}$$

The pressure loss along the sinusoids is $\Delta {p}_{h}\sim {\dot{m}}_{h}/6\times {L}_{h}/{d}_{h}^{4}$ in the case of the hexagonal shape, and $\Delta {p}_{c} \sim {\dot{m}}_{h}/4\times {L}_{h}/{d}_{c}^{4}$ in the case of the square shape. The volume of blood flowing through the lobule is a constant. Therefore we write

11 $$V=\frac{3}{2}\pi {d}_{h}^{2}{L}_{h}=\pi {d}_{c}^{2}{L}_{c}$$

And because ${L}_{c}=\frac{{3}^{3/4}}{2}{L}_{h}$, we have

12 $${\left(\frac{{d}_{h}}{{d}_{c}}\right)}^{2}={3}^{-1/4}$$

Leading to the sinusoid pressure drop ratio between a square and a hexagonal lobule:

13 $$\frac{{\Delta p}_{c}}{{\Delta p}_{h}}=\frac{3}{2}\frac{{L}_{c}}{{L}_{h}}{\left(\frac{{d}_{h}}{{d}_{c}}\right)}^{4}$$

In other words, $\Delta {p}_{h}\cong \Delta {p}_{c}$.

Finally, we conclude that the overall pressure losses will be smaller in the case of the assembly of hexagonal lobules (Eq. 9) because the mass flow rate along the ${L}_{d}$ ducts of diameter $d$ (hepatic artery and portal vein) is half the one of a square assembly. This is consistent with the results provided by Siggers et al.^40^ whose finite elements modeling in 2D indicates a reduction in blood flow rate in a square lattice as opposed to a hexagonal one.

### The porous medium approach

Scanning Electron Microscope images of human liver reveal that the elemental system, the lobule, is entirely vascularized^19^. The sinusoids tortuous network bathes the entire lobule, supplying the blood to the central vein from the 6 sources (hepatic artery + portal vein). Note that the number of 6 branches is the limit for which the radial connections exhibited in Fig. 5 is a good pattern. We demonstrated previously that beyond the value of 6 connected branches, radial networks should be replaced by tree-shaped ones with optimized diameter ratios (Eq. 3) and length ratios (Eq. 6) in order to spend less pumping power for the same fluid volume^26^.

**Figure 6:**
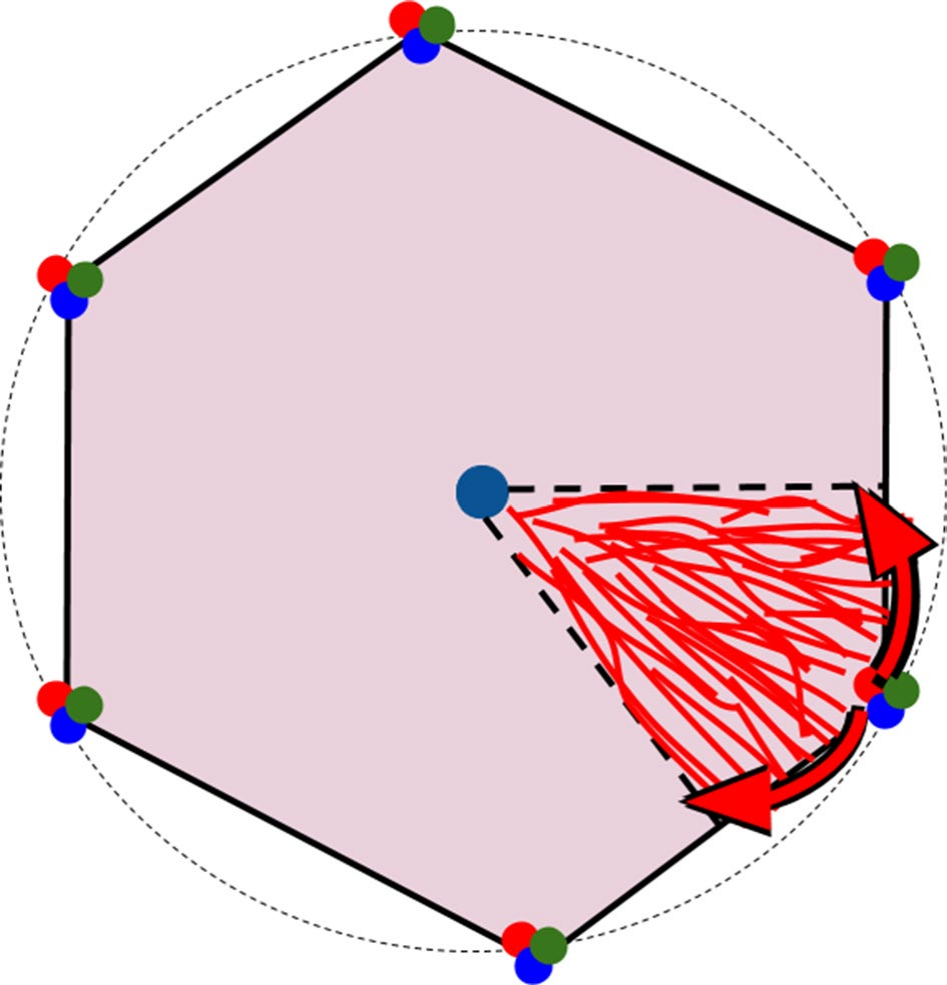
River basin in a liver lobule. The blood distribution within the lobule is similar to a reverse river basin connecting rivulets to a central sink.

We note that each source is in charge of 1/6th of the hexagonal lobule cross section. From one source, the blood not only invades the sinusoids network in the broad direction of the central vein, but it must also flow along the hexagon periphery at mid-distance from the two neighboring sources. This way, what was initially a local fluid source becomes a distributed fluid source. The sector covered has an angle of π/6 from the central vein, see Fig. 6. The entire network is similar to a river delta, except that in the case of the lobule the fluid flows in the reverse direction as in the river basin. The entire volume of the lobules is fixed because the blood volume is fixed. The network that drives the flow of blood towards the central vein is not radial as the radial design does not allow minimum friction losses^26^. At such a small scale, it seems appropriate to use a porous medium analogy^16,41–43^. The overall pressure loss from the hexagon rim to the central vein is the sum of the pressure losses along the branches of the flow dendritic pathway.

In our previous works on engineered flow architectures^26,33^, a general expression of the pressure losses was derived as a function of the fluid volume $V$ and a flow resistance factor ${f}_{n}$ that varies with the bifurcation level of the dendritic structures.

14 $$\Delta p=8\pi \nu {\dot{m}}_{h}\frac{{{L}_{h}}^{3}}{{V}^{2}}{f}_{n}$$

Here, ${L}_{h}$ is the distance between the lobule center and its perimeter.

The work dealt with a surface with a round cross section. Nevertheless considering that the hexagonal shape is close to the circle shape, the same expression was used in this work to predict the order of magnitude of the lobule permeability. The resistance factor ${f}_{n}$ is obtained from the search of minimum overall flow resistance in a laminar dendritic structure: the diameter ratio (which follows the Hess-Muray’s law^27,28^), the branches lengths and bifurcation angles correspond to minimum pressure losses. Its generic expression is

15 $${f}_{n}={n}_{0}{\left[\sum_{0}^{n}{2}^{i/3}\widehat{{L}_{i}}\right]}^{3}$$

where ${n}_{0}$ is the number of sectors connected to the source (${n}_{0}=$ 6 here), $\widehat{{L}_{i}}={L}_{i}/{L}_{h}$ the non-dimensional branch length, and n is the bifurcation level. We gathered the values of ${f}_{n}$ from Wechsatol et al.^33^ They are 13.16, 16.31, 18.67, 20.5, 21.8 and 22.6 for n = 2 to 7 respectively, which means that for increasing pairing levels, ${f}_{n}$ reaches an asymptote close to 25.

Considering Darcy flow through the porous elemental system (i.e. one lobule), the average velocity of the blood flowing to the central vein is

16 $$U=\frac{K}{\mu }\frac{\Delta p}{{L}_{h}}$$

where *K* is the intrinsic permeability of the lobule.

The mass flow rate is related to the average velocity through ${\dot{m}}_{h}=6\rho U{L}_{h}t$, with *t* the lobule thickness. Finally, the permeability of a lobule of volume $V$ is

17 $$K\sim \frac{{V}^{2}}{48\pi {{t L}_{h}}^{3}{f}_{n}}$$

which, in view of the asymptotic value of ${f}_{n}$, gives

18 $$K\cong 2.6 1{0}^{-4}\frac{{V}^{2}}{{{t L}_{h}}^{3}}$$

According to the literature^15,44,45^, the average human liver has a volume of 1500 cm^3^, and contains 10–20% of blood, while it possesses about 10^6^ lobules. This would give a lobule volume of 1.5 mm^3^. On another hand, Debbaut et al.^46^ reported a value of 0.134 mm^3^ for 3 human liver lobules. The lobules dimensions are also difficult to find in the literature. They are reported to range from 500 µm up to 2.5 mm in diameter for humans^14,42,47^. In the absence of more precise data, Eq. 18 gives a permeability K ranging between 3 10^–10^ m^2^ and 9 10^–12^ m^2^. This result is in agreement with the literature as the radial and tangential permeability of a lobule were estimated to be about 1.5 10^–14^ m^2^ in Ref^46^, while Ref^11^. reports a lobule permeability of 4.8 10^–9^ m^2^.

## Conclusion

The objective of this work was to propose a framework aiming at predicting the hepatic blood circulation. We showed that the entire liver circulatory architecture is deterministic. We deconstructed the liver as a canopy-to-canopy architecture of trees made of 2 trees running in parallel (the hepatic artery and the portal vein) combined with the hepatic vein tree. The three tree-shaped architectures correspond to a volume-to-point flow. This canopy-to-canopy feature is complemented by a porous medium, the lobules. Thanks to the constructal law we could predict the main geometrical features of the liver:

1. The diameter ratio of each tree has a unique value and obeys the Hess-Murray’s law,
2. The channels length ratio of each tree is similar to the diameter ratio and follows the equipartition of thermodynamic imperfections principle,
3. The microcirculation happens through lobules which hexagonal shape corresponds to minimum flow resistances,
4. The blood transport through the lobules behaves like a flow through a porous system which predicted overall permeability agrees with data available in the literature.

This work establishes the theoretical bases that help to complete the understanding of the results of experimental work carried out since the last century on animal models, as well as the most recent ones on experimental models on silicon scaffolds, organs on chips, and scaffolds of decellularized organs. Such theoretical framework may be useful in the design of perfusion models both at micro and macro levels on the way to perfecting a functional prediction in the new coordinated and multidisciplinary efforts of regenerative medicine between other multiple physical scenarios.

## Data Availability

The data will be available upon request.
